# Supplementary material for: Nonlinear Relationships among the Natural Environment, Health, and Sociodemographic Characteristics across US Counties
Source: Int J Environ Res Public Health. 2022 Jun 4;19(11):6898. doi: 10.3390/ijerph19116898 (PMC9180717; doi:10.3390/ijerph19116898)
Supplement: Supplementary file 1 [file ijerph-19-06898-s001.zip › ijerph-1715435-supplementary.pdf]

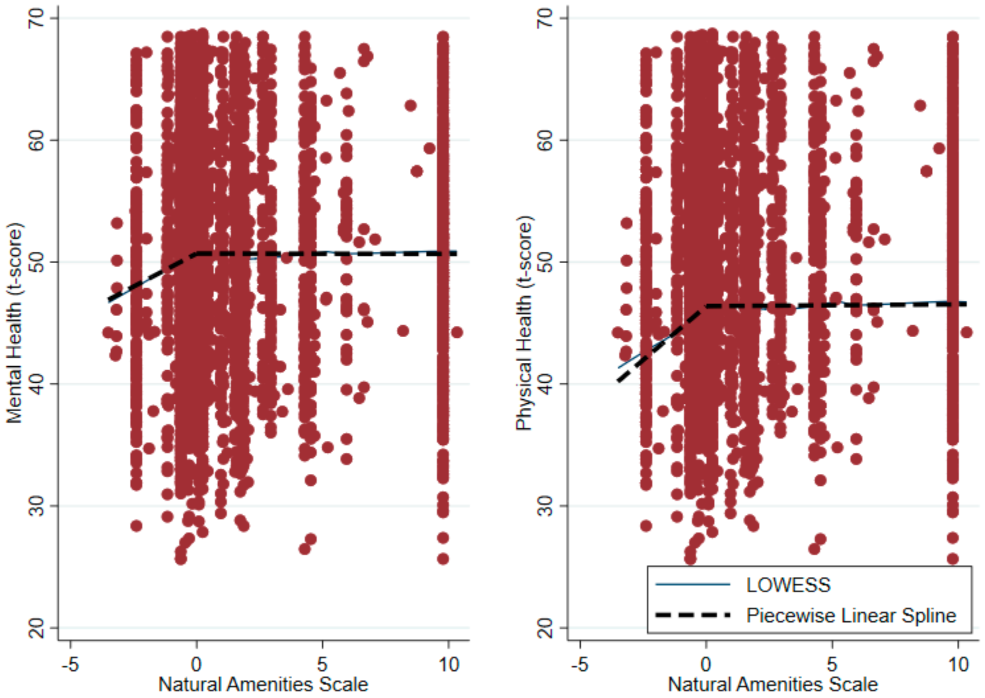

Supplementary Figure S1. LOWESS curves, piecewise linear splines, and data points visualizing the unadjusted relationships among NAS and mental and physical health.

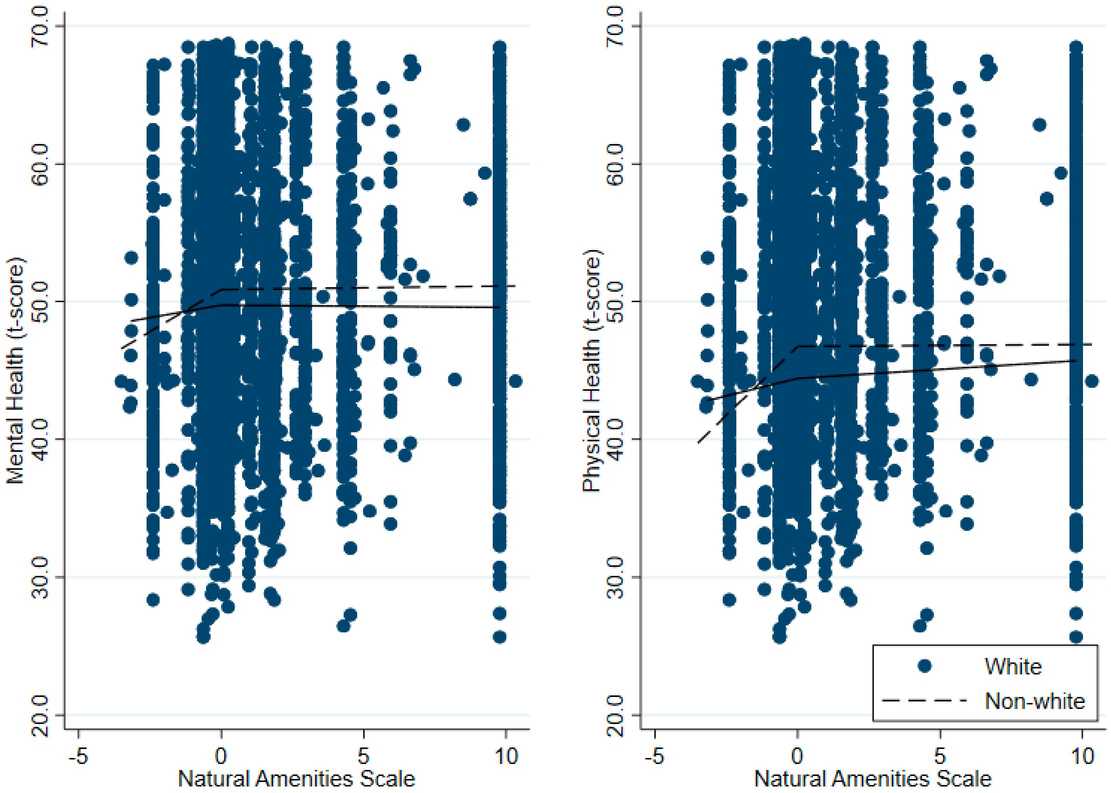

Supplementary Figure S2. Unadjusted piecewise linear spline models visualizing the relationships between NAS and mental and physical health for white (gray lines) and non-white (black lines) individuals.
